# Comparative persistence of antiepileptic drugs in patients with epilepsy: A STROBE-compliant retrospective cohort study

**DOI:** 10.1097/MD.0000000000004481

**Published:** 2016-09-02

**Authors:** Edward Chia-Cheng Lai, Cheng-Yang Hsieh, Chien-Chou Su, Yea-Huei Kao Yang, Chin-Wei Huang, Swu-Jane Lin, Soko Setoguchi

**Affiliations:** a School of Pharmacy, Institute of Clinical Pharmacy and Pharmaceutical Sciences, College of Medicine; b Duke Clinical Research Institute, Duke University School of Medicine, Durham, North Carolina; c Department of Neurology, Tainan Sin Lau Hospital; d Health Outcome Research Center; e Department of Neurology, National Cheng Kung University Hospital, Tainan, Taiwan; f Department of Pharmacy Systems, Outcomes & Policy, College of Pharmacy, University of Illinois at Chicago, Chicago, Illinois.

**Keywords:** Antiepileptic drugs, Comparative effectiveness research, Epilepsy, Persistence, Treatment retention

## Abstract

We compared persistence of antiepileptic drugs (AEDs) including carbamazepine, oxcarbazepine, gabapentin, lamotrigine, topiramate, valproic acid, and phenytoin in an Asian population with epilepsy.

A retrospective cohort study was conducted by analyzing Taiwan's National Health Insurance Research Database (NHIRD). Adult epilepsy patients newly prescribed with AEDs between 2005 and 2009 were included. The primary outcome was persistence, defined as the treatment duration from the date of AED initiation to the date of AED discontinuation, switching, hospitalization due to seizure or disenrollment from databases, whichever came first. Cox proportional hazard models were used to estimate the risk of non-persistence with AEDs.

Among the 13,061 new users of AED monotherapy (mean age: 58 years; 60% men), the persistence ranged from 218.8 (gabapentin) to 275.9 (oxcarbazepine) days in the first treatment year. The risks of non-persistence in patients receiving oxcarbazepine (adjusted hazard ratio [HR], 0.78; 95% CI, 0.74–0.83), valproic acid (0.88; 0.85–0.92), lamotrigine (0.72; 0.65–0.81), and topiramate (0.90; 0.82–0.98) were significantly lower than in the carbamazepine group. Compared with carbamazepine users, the non-persistence risk was higher in phenytoin users (1.10; 1.06–1.13), while gabapentin users (1.03; 0.98–1.09) had similar risk. For risk of hospitalization due to seizure and in comparison with carbamazepine users, oxcarbazepine (0.66; 0.58–0.74) and lamotrigine (0.46; 0.35–0.62) users had lower risk, while phenytoin (1.35; 1.26–1.44) users had higher risk. The results remained consistent throughout series of sensitivity and stratification analyses.

The persistence varied among AEDs and was better for oxcarbazepine, valproic acid, lamotrigine, and topiramate, but worse for phenytoin when compared with carbamazepine.

## Abstract 2

Supplemental Digital Content is available in the text

## 1 Introduction

Antiepileptic drugs (AEDs) are the mainstay of treatment for patients with epilepsy; however, management of epilepsy with AEDs can be complicated due to several factors, including concurrent medical comorbidities, drug interactions, and long-term side effects.^[1]^ A global assessment of patients and clinical consideration should be made when selecting an AED regimen for patients with epilepsy.^[2]^ Persistence, namely treatment retention, represents a patient's consistency in staying with a prescribed regimen and integrates the assessments of patients and clinicians regarding efficacy, safety, and tolerability into a composite measurement of effectiveness.^[3–6]^ Persistence reflects therapeutic benefits in relation to undesirable effects, and has been used as primary outcome in many clinical trials and observational studies.^[3–10]^ It is also a primary outcome measure recommended by the Commission on Antiepileptic Drugs of the International League Against Epilepsy in evaluating AED effectiveness.^[3,6,7]^

The Standard and New Antiepileptic Drugs (SANAD) trial was a pragmatic randomized controlled trial that was very close to clinical practice, with participation of a large number of primary care physicians as well as neurologists.^[4,5]^ It maintained the flexibility for the prescriber to adjust doses and change medications as required. By evaluating the persistence of AEDs, the SANAD concluded that the time to treatment failure of lamotrigine was better than topiramate in patients with generalized epilepsy and unclassifiable epilepsy,^[5]^ and was also better than carbamazepine in patients with partial epilepsy.^[4]^ However, the generalizability of results from SANAD to the Asian population is unknown, because the polymorphisms of hepatic enzymes among the Asian population differ from those of the Caucasian^[11,12]^ and may result in a considerable difference in plasma concentration and effect profiles of AEDs.^[13–16]^ Thus, we compared persistence, the same primary outcome measurement of SANAD, among six different AEDs in Taiwanese patients with epilepsy using a large nationwide database compiled from real-world clinical practice data.

## 2 Method

### 2.1 Data source

The National Health Insurance Research Database (NHIRD) of Taiwan was used in this study.^[17]^ Taiwan launched a single-payer mandatory National Health Insurance (NHI) program on March 1, 1995, and the entire population of Taiwan (approximately 23.16 million individuals) was enrolled. The National Health Research Institute (NHRI) compiles information, including demographics of enrollees, characteristics of healthcare professionals and facilities, and service claims from inpatient, ambulatory-care and contracted pharmacies from insurance claims, for research purposes.^[17]^ The accuracy of some major disease diagnoses in the NHIRD, such as epilepsy, stroke, renal dysfunction, acute coronary syndrome, and myocardial infarction, has been validated.^[18–22]^ Because all AEDs are reimbursed by the NHI program, the entirety of prescriptions were captured by NHIRD, providing a good opportunity to evaluate the persistence of AEDs. All researchers using the NHIRD are required to sign a written declaration that they will not attempt to obtain information that could violate patients’ or care providers’ privacy, and will respect the Taiwan Personal Information Protection Act. The use of NHIRD data, without cross-linkage to external health data, required the approval of NHRI and was exempted from Institutional Review Board review in Taiwan.^[17]^

### 2.2 Study population

A retrospective cohort study was conducted including epilepsy patients aged 18 and older who initiated monotherapy with carbamazepine, phenytoin, oxcarbazepine, valproic acid, topiramate, lamotrigine, or gabapentin between 2005 and 2009. To ensure that the AED was prescribed as long-term antiepileptic treatment, we selected patients who had received the targeted AED for ≥90 days. The cutoff of 90 days was based on previous studies of antiepileptic therapy.^[23,24]^ Epilepsy has been identified by the International Classification of Diseases, Ninth Revision, Clinical Modification (ICD-9-CM) with a code of 345.xx, and further classified into two subtypes, generalized or unspecified epilepsy (ICD-9-CM code 345.0, 345.1, 345.2, 345.3, and 345.9) and partial epilepsy (ICD-9-CM code 345.4, 345.5, 345.7). The accuracy of epilepsy diagnosis in the NHIRD was previously shown to have a sensitivity and specificity of 83.91% and 99.83%, respectively.^[19]^ Patients were categorized into AED groups according to the first AED prescribed, and the first prescription date was defined as the index date. Patients not receiving any AED prescription within 3 years prior to index date were considered new users. Patients with NHI eligibility less than 3 years before the index date were excluded to ensure sufficient data to confirm patients’ status as incident users.

### 2.3 Definition of outcome and treatment changes

The comparative effectiveness among AEDs was evaluated by the persistence with the medications during the first year of treatment, where persistence was defined as the treatment duration from the index date to the earliest date of switching to or augmenting with another AED, discontinuation of AED (no dispensing for >90 days after the end date of the previous dispensing), hospitalization due to seizure, or disenrollment from NHI, whichever came first. The 90-day period between two prescriptions was adopted on the basis of previous reports, indicating gaps rarely exceeded 90 days without complete or long-term discontinuation of a medication.^[8,25]^ Death was the most common reason for disenrollment from NHI.

### 2.4 Statistical analysis and covariates

We chose carbamazepine as the reference group because carbamazepine was the most-prescribed agent throughout major developed countries at the time of this study.^[26–28]^ Kaplan–Meier survival curves were used to evaluate persistence of AEDs. Regression based on Cox proportional hazards model with outcome indicator of time to non-persistence was used to compare among AEDs. We collected information in a 1-year baseline period before the index date to adjust for differences between AED groups. Approximately, 70 potential confounding covariates based on previously published studies were included in the regression models,^[24]^ such as patient demographics (i.e., age, sex, NHI premium levels), AED dosages, index year, accreditation level of hospitals at index date, comorbidities, and concomitant medications. AED relative dosages were calculated by two indicators, defined daily dose (DDD) and prescribed daily dose (PDD). The DDD assignment was based on dose information from the World Health Organization Collaborating Center and the PDD was calculated from prescription data of hospital visits.^[29]^ Thus, the PDD/DDD ratio of an AED indicates the relative dosage of what has been prescribed as compared with what has been recommended. Details of covariates are listed in Table 1.

**Table 1:**
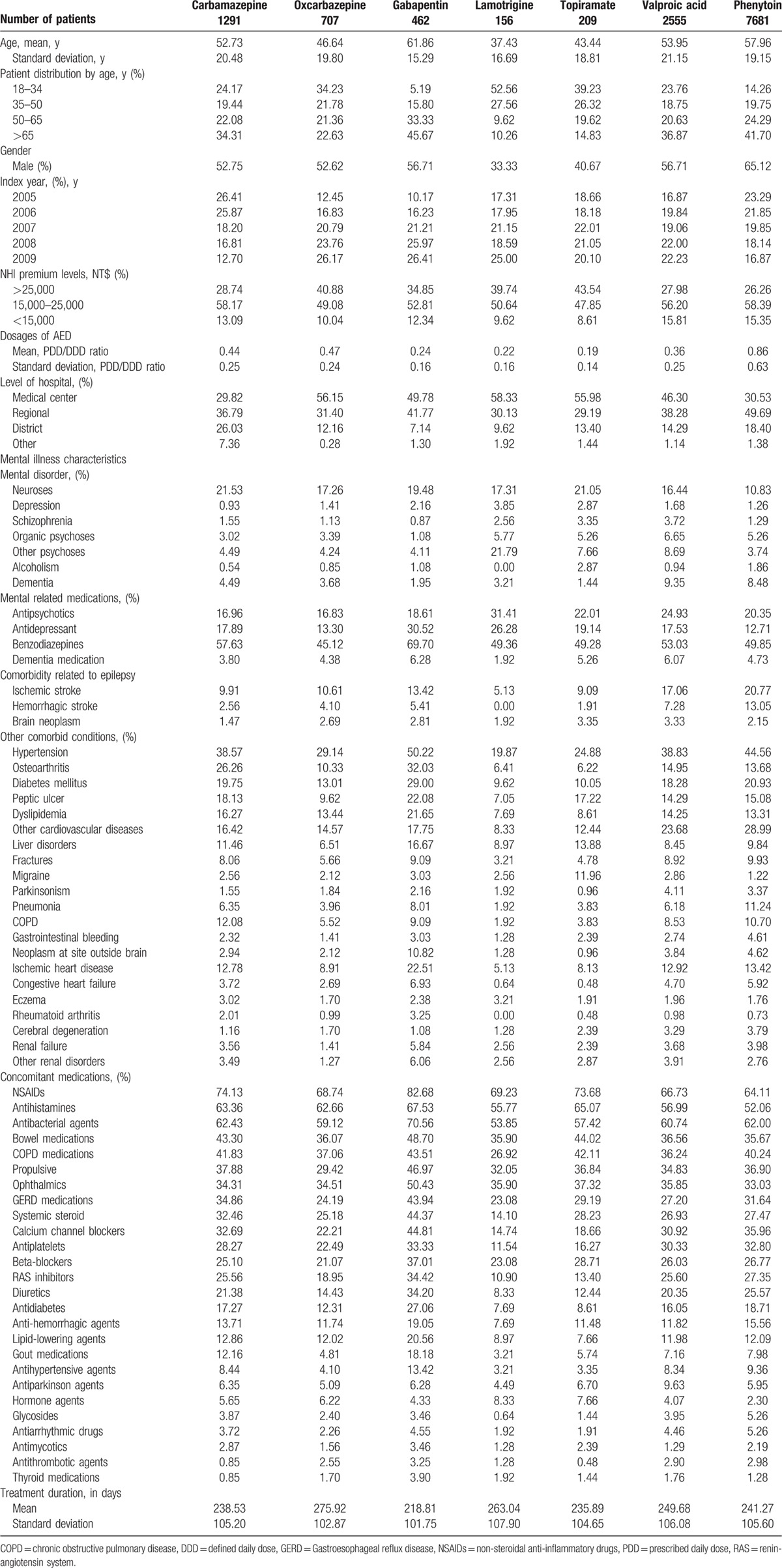
Patient characteristics of antiepileptic drug users.

### 2.5 Subgroup and sensitivity analyses

We used the high dimensional propensity score (hdPS) method^[30]^ to estimate the conditional probability of a patient being prescribed with carbamazepine versus one of the other AEDs. Similar to the more commonly used techniques of matching or inverse probability weighting, the goal of hdPS is to minimize bias in non-experimental studies by aggregating covariates into a summarized measure.^[30–32]^ We used published algorithms to screen the NHI data to identify covariates that might be relevant.^[30,32,33]^ Inverse probability weighting approach with propensity score (hdPS-IPW) was performed to balance potential differences between the treatment groups.^[31]^ We implemented hdPS-IPW to create a pseudo-population with two treatment groups of similar characteristics by weighting the inverse probability of each patient receiving carbamazepine or one of the other AEDs. Compared with matching, the weighting approach does not require study sample constraint, thus has better generalizability.^[31]^ The adjusted outcome estimation represents the average expected treatment effect of the two comparable patient groups. Additionally, carbamazepine was matched in a 1:1 ratio to patients using the other AEDs, by using the Greedy 5 to 1 digit technique based on hdPS.^[34]^

We extracted two sub-cohorts with more homogenous patients to evaluate the robustness of the main results: Subgroup 1 included patients without mental disorder diagnosis in the study period. Subgroup 2 included patients without catastrophic illness certificate. Patients considered as having a catastrophic illness, as defined by Bureau of National Health Insurance, Taiwan, are exempt from NHI copayments. The catastrophic illness status indicates the severity of a patient's baseline health status.^[17]^ We repeated our analyses with various treatment period lengths (0, and 30 days) other than the 90-day requirement of targeted AED treatment in the main analysis. We also repeated our analyses with various lengths (0 and 30 days) of discontinuation other than the 90-day period in the main analysis. Furthermore, persistence was analyzed separately for the four causes of non-persistence (i.e., discontinuation, switching/augmenting, hospitalization due to seizure, and disenrollment). Hospitalization due to any cause was also evaluated in the sensitivity analysis to test the results of this indicator for non-persistence.

A last set of sensitivity analyses stratified patients by subtypes of epilepsy (generalized or unspecified epilepsy; partial epilepsy), age (elderly, ≥65; young adult, 18–64), sex, level of hospital (medical center; non-medical center), and relative dosage (by PDD/DDD ratio ≥0.5 and <0.5) of AEDs to evaluate the relative effects. All aforementioned sub-analyses and sensitivity analysis are summarized in Table 3. All statistical analyses were performed using SAS 9.3 version software (SAS Institute, Cary, NC).

## 3 Results

The datasets of a total of 17,743 individuals diagnosed with epilepsy and newly receiving monotherapy with targeted AEDs between 2005 and 2009 were retrieved from the NHIRD. We excluded 225 patients without 3 years enrollment in NHI prior to the index date and 4457 patients younger than 18 years at the index date. After the exclusions, 13,061 patients remained as the study cohort. Of this cohort, 1291 received carbamazepine, 7681 received phenytoin, 2555 received valproic acid, 707 received oxcarbazepine, 462 received gabapentin, 209 received topiramate, and 156 received lamotrigine. Patients’ baseline characteristics for the groups are presented in Table 1. To summarize, the gabapentin group had a higher proportion of elderly patients aged 65 and older (22.63%), and a higher rate of comorbidities and co-medications, including hypertension (50.22%), diabetes mellitus (29.00%), dyslipidemia (21.65%), ischemic heart disease (22.51%), and the use of antidepressants (30.52%), benzodiazepines (69.70%), beta-blockers (37.01%), calcium channel blockers (44.81%), and lipid-lowering agents (20.56%); while the lamotrigine group had a lower proportion of elderly patients (10.26%) and a lower rate of comorbidities and co-medications compared with other groups.

**Figure 1:**
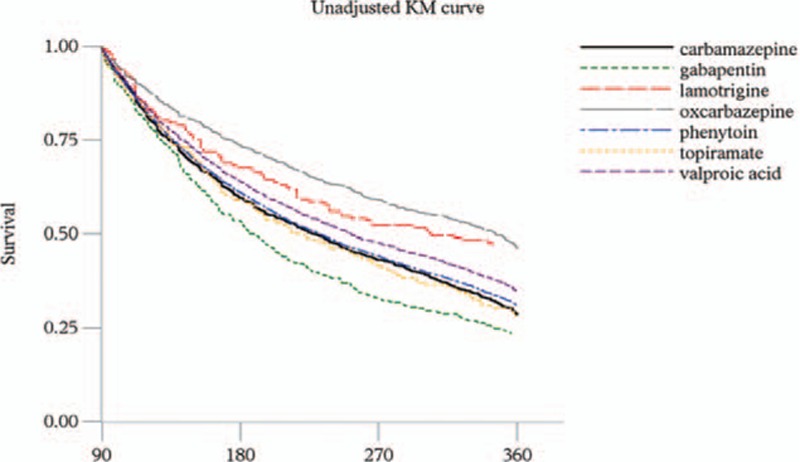
Kaplan–Meier survival curves of persistence comparisons among antiepileptic drugs.

Approximately, 74% of carbamazepine, 71% of phenytoin, 67% of valproic acid, 56% of oxcarbazepine, 78% of gabapentin, 75% of topiramate, and 54% of lamotrigine treated patients became non-persistent within 1-year of initiation of AEDs. The mean treatment duration ranged from 218.8 days (gabapentin) to 275.9 days (oxcarbazepine) in the first treatment year. Results from the regression models showed that, compared with the carbamazepine group, the risk of non-persistence was significantly lower in patients receiving oxcarbazepine (adjusted hazard ratio [HR], 0.78; 95% CI, 0.74–0.83), valproic acid (0.88; 0.85–0.92), lamotrigine (0.72; 0.65–0.81), or topiramate (0.90; 0.82–0.98), while the risk was significantly higher among patients receiving phenytoin (1.10; 1.06–1.13) (Table 2). Other risk factors for non-persistence were age of 65 or older (1.06; 1.03–1.10), higher dosages of AED (1.17; 1.16–1.19 per PDD/DDD ratio), the presence of pneumonia, chronic obstructive pulmonary disease, congestive heart failure, renal failure, gastrointestinal bleeding, and the use of propulsives, diuretics, systemic steroid agents, nonsteroidal anti-inflammatory drugs, and benzodiazepines. The Kaplan–Meier survival curves comparing treatment persistence with AEDs over time are shown in Fig. 1. The unadjusted curves indicate that oxcarbazepine has the lowest risk of non-persistence within 1 year of AED initiation, followed by lamotrigine, valproic acid, phenytoin, carbamazepine, topiramate, and gabapentin.

**Table 2:**
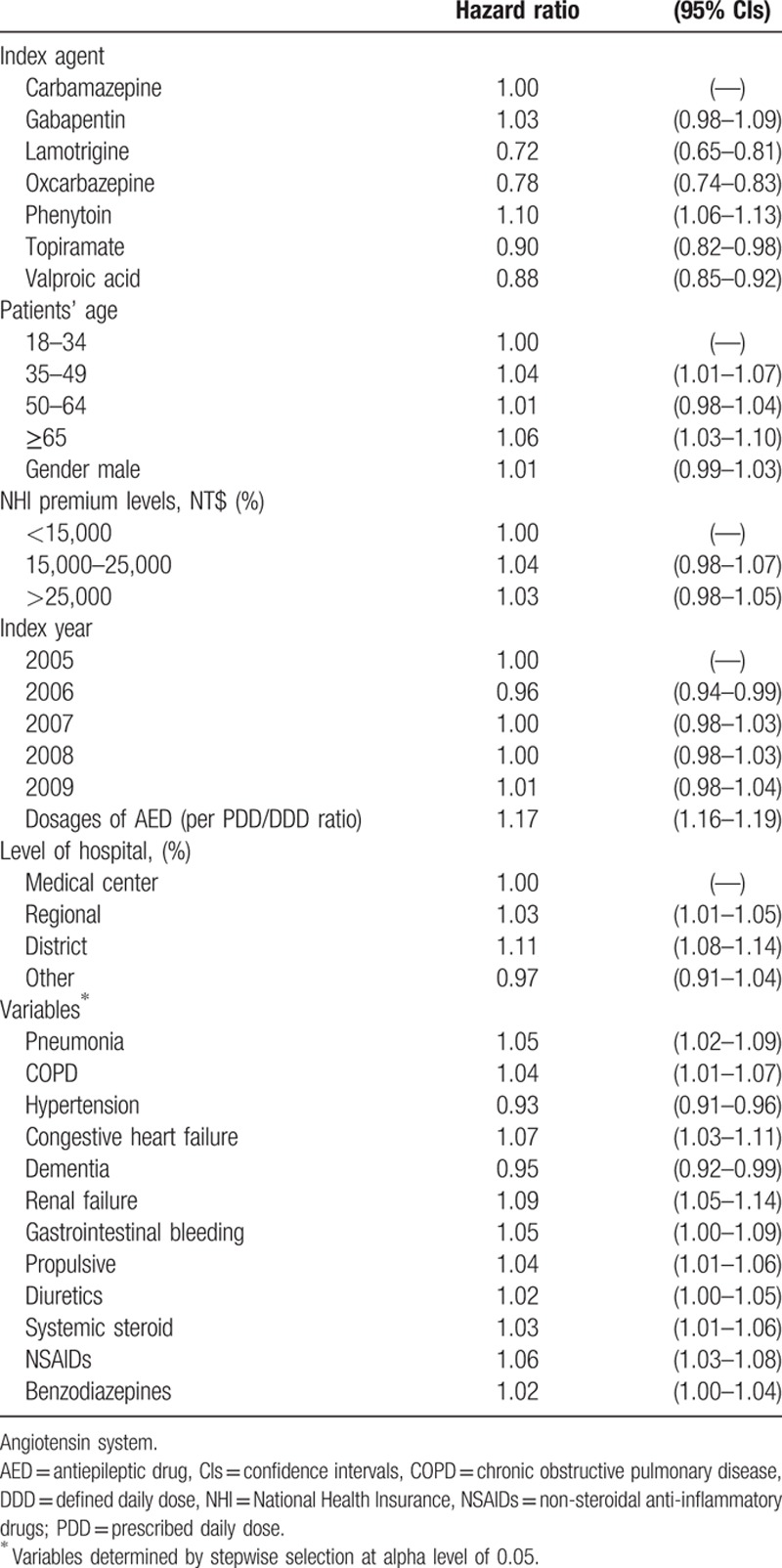
Multivariate Cox proportional hazards regression model for risk of non-persistence.

### 3.1 Subgroup and sensitivity analyses

After adjustment with hdPS-IPW and matching, we generated cohorts with balanced baseline characteristics for comparison. (Appendix Table 1 and 2). Results from hdPS-IPW and hdPS matching were largely similar to results from multivariate regression models. Of all patients with treatment changes, 57% discontinued treatment, 31% switched or added another AED, 11% were hospitalized due to seizure relapse, and 1% was censored due to disenrollment within 1-year of treatment. Hazard ratios for specific causes of non-persistence were similar to those of all causes of non-persistence, indicating a significantly lower risk for oxcarbazepine, valproic acid, lamotrigine, and topiramate, but not phenytoin and gabapentin, when compared with carbamazepine. Specifically, oxcarbazepine (0.66; 0.58–0.74) and lamotrigine (0.46; 0.35–0.62) users had a lower risk of hospitalization due to seizure, while phenytoin (1.35; 1.26–1.44) users had a higher risk of hospitalization due to seizure or any cause when compared with carbamazepine. Users of oxcarbazepine and lamotrigine had lower risks of non-persistence in both subtypes of epilepsy. Users of valproic acid, gabapentin, and topiramate had lower risks in generalized/unspecified epilepsy, but comparable risks of non-persistence to carbamazepine in patients with partial epilepsy (valproic acid, 1.00; 0.89–1.12; gabapentin, 0.93; 0.78–1.09; topiramate 0.98; 0.80–1.19). The results from subgroups and several sensitivity analyses were generally consistent with the main results where valproic acid, oxcarbazepine, topiramate, and lamotrigine users tended to have lower risks, while phenytoin and gabapentin had higher risks of non-persistence when compared with carbamazepine users (Table 3 and Appendix Fig. 1).

**Table 3:**
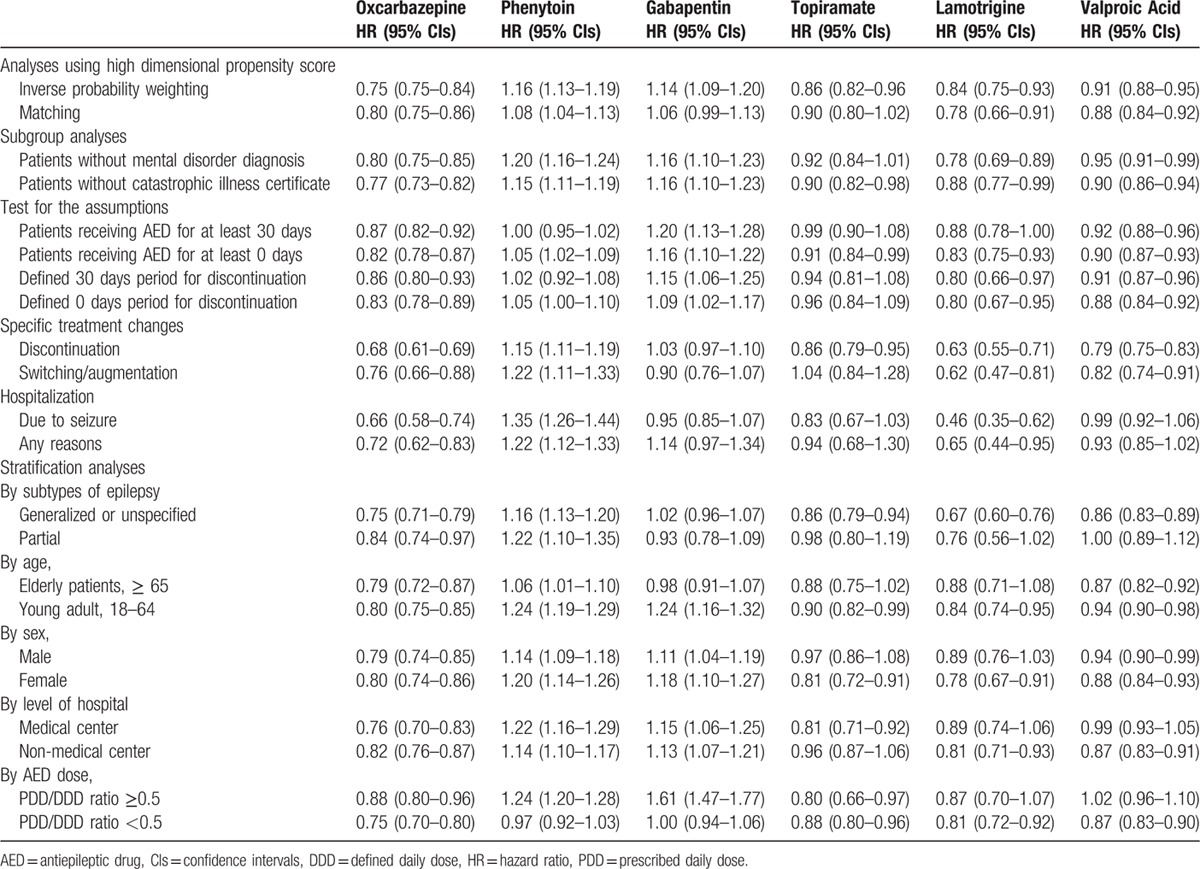
Subgroup analyses and sensitivity analyses of risk of non-persistence compared with carbamazepine user.

## 4 Discussion

We found that effectiveness of AED, as evaluated by persistence, was significantly better among oxcarbazepine, valproic acid, lamotrigine, and topiramate users, but significantly worse among phenytoin users when compared with carbamazepine in Taiwanese patients with epilepsy. Specifically, oxcarbazepine and lamotrigine users had lower risks of hospitalization due to seizure, while, in contrast, phenytoin users had higher risk of hospitalization due to seizure compared with carbamazepine users.

Although it was difficult to extract the actual reasons for non-persistence from the claims database, some insight could be inferred from the patterns of non-persistence we observed. For example, a possible reason for discontinuation could be related to seizure itself or side effects from the treatment, or both; switching to another AED could reflect a clinician's attempt to balance the trade-off between harm and benefits of a treatment, while a hospitalization reflected a lack of effective seizure control under the AED therapy.^[10]^ The significant likelihood of oxcarbazepine, valproic acid, lamotrigine, and topiramate over carbamazepine in achieving treatment persistence was found in every type of non-persistence in the current study, and the robustness of the results was confirmed by series of sensitivity analyses.

We found there was an increase in the use of later generation AEDs as initial therapy, including oxcarbazepine, gabapentin, lamotrigine, and topiramate from 2005 to 2009, while the use of carbamazepine and phenytoin were decreased. One possible explanation for this trend is that some clinicians might have perceived that the newer AEDs, with potentially lower risks of drug interactions and serious adverse skin reactions but similar efficacy for seizure control as compared with older AEDs, might be a good alternative for vulnerable populations, such as elderly patients.^[2,35,36]^ Additionally, we found a higher proportion of patients receiving later generation AEDs at medical centers than at other levels of healthcare facilities. This might be because there are more specialized neurologists in medical centers in Taiwan, who usually prefer to prescribe later generation AEDs compared with general physicians.^[23]^ Our findings support previous reports that gabapentin was widely used for newly onset geriatric epilepsy, possibly because gabapentin has an advantageous pharmacokinetic profile such as the lack of hepatic metabolism and protein binding.^[23,37]^ Furthermore, fewer female patients used phenytoin, possibly out of concern over cosmetic side effects of phenytoin such as gingival hyperplasia.^[38]^

Some of the findings in the study were consistent with the SANAD trials.^[4,5]^ For example, we found lamotrigine to have better persistence than carbamazepine in patients with partial epilepsy, and a trend that lamotrigine had better persistence than valproic acid and topiramate in generalized epilepsy. However, we found that the non-persistence rate was lower in SANAD (approximately 40%)^[4]^ than in our study (approximately 60%) within the first year after AED initiation. Although SANAD is a pragmatic RCT and very similar to the real world setting, patients were still subject to extra attention from the participating physicians. As a result, even with a similar side-effect burden, patients may have been more likely to persist on AEDs in clinical trial settings (SANAD) than in our study. Two recent Western studies using insurance claims data also demonstrated a low persistence rate in epileptic patients after initiating an AED therapy.^[39,40]^

Moreover, while we found that oxcarbazepine had better persistence than carbamazepine in patients with partial epilepsy, the two AEDs showed similar persistence in SANAD; similarly, while we found gabapentin to be comparable to carbamazepine, it was shown to be worse than carbamazepine in patients with partial epilepsy in SANAD. Our patients differ from those of previous RCTs in baseline characteristics, for example, greater age, more comorbidities, and co-medications. We speculated that certain patient-level factors such as polypharmacy, drug interaction, and poor tolerability might reduce the efficacy of older AEDs in our patients. A recent Taiwanese study^[41]^ also found that, for patients with post-stroke epilepsy, the efficacy was better with valproic acid and newer AEDs than with older AEDs like phenytoin. Their findings were comparable to ours, despite the differences in the study population and the definition of efficacy.

We found that higher prescribed dosage of AED was associated with higher risk of non-persistence (1.17; 1.16–1.19 per PDD/DDD ratio). Presumably, patients receiving higher doses might have higher risk of side effects, leading to higher possibility of AED discontinuation or switching. The higher prescribed dosage of AED might also reflect a higher baseline disease severity in those epilepsy patients, which cannot be determined from claims data but is an important determinant of persistence on AEDs. Several risk factors for non-persistence were also identified, including age, comorbidities, and certain co-medications; such information might be useful for clinicians when prescribing AEDs for their patients with epilepsy.

### 4.1 Strengths and limitations

Using a large nationwide dataset representative of Taiwan's entire population was the strength of the current study. Because all AEDs were reimbursed as medical therapy for epilepsy under Taiwan's NHI system, we were able to capture all AED use by patients in the study cohort, which increased accuracy of the AED persistence measure. Additionally, since AED costs were not of concern to patients, the persistence of AEDs was not affected by economic considerations on the patient's or physician's part. We included only new users in this study in an attempt to create a relatively and comparatively homogenous cohort. Because epilepsy diagnosis in NHIRD had been previously validated,^[19]^ the validity of the diagnosis was ensured. Moreover, many potential confounders were considered, and results remained consistent throughout the weighted processes with hd-PS technique, as well as the series of sensitivity and stratification analyses. The results were shown to be robust.

As in all observational studies using electronic databases, we were unable to confirm whether patients actually took the dispensed medications. Presumably, all treatment changes identified in this study reflected assessments and decisions by physicians or patients themselves as a result of poor drug adherence and unsatisfactory outcomes such as hospitalization due to seizure episode. However, we could not rule out that some physicians might consider the newer generation of AEDs to be better than carbamazepine and were more reluctant to switch or augment the newer AEDs. Although a 3-year observational window of no-treatment prior to the index date was set to ensure that the selected patients were new users, this study might have included some patients who had actually used AEDs more than 3 years prior to the index date; however, the likelihood is low if a patient was indeed diagnosed with epilepsy. The NHIRD does not have information on baseline disease severity. To adjust the potential effect of confounding by indication, several analyses were performed by creating relatively homogenous cohorts, and the consistency of results indicates the robustness of the study. Therefore, the potential bias due to these limitations is unlikely to have a significant impact on the study results. Several inclusion and exclusion criteria were used to increase the internal validity of the study; however, this might have limited the external validity of this study, for example, pediatric patients were not discussed in this study.

## 5 Conclusion

The present study indicated that the persistence was varied among AEDs, with higher persistence among oxcarbazepine, valproic acid, lamotrigine, and topiramate users, and lower among phenytoin users when compared with carbamazepine users in an Asian population with epilepsy.

## Supplementary Material

Supplemental Digital Content
