# Supplementary material for: Comparative persistence of antiepileptic drugs in patients with epilepsy: A STROBE-compliant retrospective cohort study
Source: Medicine (Baltimore). 2016 Sep 2;95(35):e4481. doi: 10.1097/MD.0000000000004481 (PMC5008541; doi:10.1097/MD.0000000000004481)

| **Appendix Table1.** Patient characteristics by inverse probability weighting with high-dimensional propensity score | | | | | | | | | | | | |
| --- | --- | --- | --- | --- | --- | --- | --- | --- | --- | --- | --- | --- |
|  | **Cohort with IPW-hdPS** | | | | | | | | | | | |
|  | **IPW Cohort 1** | | **IPW Cohort 2** | | **IPW Cohort 3** | | **IPW Cohort 4** | | **IPW Cohort 5** | | **IPW Cohort 6** | |
|  | **CBZ** | **OXC** | **CBZ** | **GBP** | **CBZ** | **LTG** | **CBZ** | **TPM** | **CBZ** | **VPA** | **CBZ** | **PHT** |
| Number of patients | 1291 | 707 | 1291 | 462 | 1291 | 156 | 1291 | 209 | 1291 | 2555 | 1291 | 7681 |
| Age, mean , y | 50.59 | 50.79 | 54.96 | 56.33 | 51.45 | 47.33 | 51.42 | 45.66 | 52.04 | 53.30 | 57.02 | 57.37 |
| Standard deviation, y | 20.39 | 20.54 | 19.62 | 18.11 | 20.51 | 22.16 | 20.55 | 19.03 | 20.65 | 21.11 | 19.91 | 19.47 |
| Patient distribution by age, y (%) |  |  |  |  |  |  |  |  |  |  |  |  |
| 18-34 | 27.40 | 27.60 | 19.28 | 18.05 | 26.81 | 36.60 | 26.45 | 41.91 | 25.60 | 24.62 | 16.56 | 15.78 |
| 35-50 | 20.70 | 20.62 | 17.89 | 17.23 | 19.87 | 23.25 | 20.00 | 18.47 | 19.24 | 19.30 | 18.98 | 19.60 |
| 50-65 | 21.68 | 20.59 | 25.30 | 28.49 | 20.84 | 10.95 | 21.70 | 20.01 | 22.28 | 20.61 | 22.06 | 23.89 |
| >65 | 30.23 | 31.18 | 37.53 | 36.24 | 32.47 | 29.20 | 31.85 | 19.61 | 32.88 | 35.47 | 42.40 | 40.73 |
| Gender |  |  |  |  |  |  |  |  |  |  |  |  |
| Male (%) | 53.57 | 53.86 | 52.37 | 57.27 | 51.88 | 47.90 | 49.63 | 43.38 | 53.51 | 55.48 | 59.22 | 63.26 |
| Index year, (%), y |  |  |  |  |  |  |  |  |  |  |  |  |
| 2005 | 21.77 | 18.02 | 21.95 | 14.33 | 25.42 | 12.17 | 26.34 | 25.49 | 20.20 | 19.91 | 25.23 | 24.14 |
| 2006 | 22.71 | 24.52 | 23.39 | 21.93 | 25.38 | 25.81 | 25.71 | 15.79 | 22.61 | 22.02 | 31.72 | 22.02 |
| 2007 | 19.47 | 19.84 | 18.33 | 21.99 | 18.33 | 15.24 | 17.61 | 24.16 | 18.76 | 18.54 | 17.45 | 19.47 |
| 2008 | 19.32 | 20.21 | 20.30 | 23.65 | 16.86 | 22.00 | 17.11 | 12.18 | 20.43 | 20.15 | 15.31 | 17.67 |
| 2009 | 16.74 | 17.41 | 16.03 | 18.10 | 14.00 | 24.77 | 13.23 | 22.38 | 18.00 | 19.38 | 10.29 | 16.71 |
| NHI premium levels, NT$ (%) |  |  |  |  |  |  |  |  |  |  |  |  |
| >25,000 | 30.78 | 38.30 | 28.86 | 34.09 | 27.97 | 30.36 | 29.14 | 34.40 | 28.95 | 27.69 | 28.05 | 26.04 |
| 15,000-25,000 | 56.47 | 46.74 | 58.35 | 55.62 | 59.46 | 53.93 | 58.73 | 53.57 | 58.26 | 56.64 | 58.55 | 58.57 |
| <15,000 | 12.75 | 14.96 | 12.79 | 10.29 | 12.56 | 15.72 | 12.13 | 12.03 | 12.79 | 15.68 | 13.40 | 15.39 |
| Dosages of AED, |  |  |  |  |  |  |  |  |  |  |  |  |
| Mean, PDD/DDD ratio | 0.44 | 0.45 | 0.39 | 0.36 | 0.43 | 0.40 | 0.41 | 0.36 | 0.40 | 0.40 | 0.65 | 0.81 |
| Standard deviation, PDD/DDD ratio | 0.25 | 0.22 | 0.24 | 0.23 | 0.25 | 0.26 | 0.25 | 0.22 | 0.23 | 0.41 | 0.31 | 0.61 |
| Level of hospital, (%) |  |  |  |  |  |  |  |  |  |  |  |  |
| Medical center | 37.71 | 39.52 | 34.96 | 35.57 | 32.34 | 34.38 | 34.10 | 36.77 | 38.38 | 40.38 | 28.73 | 30.29 |
| Regional | 36.10 | 32.62 | 38.10 | 41.41 | 35.77 | 30.37 | 35.35 | 50.21 | 39.45 | 38.01 | 33.47 | 47.21 |
| District | 21.28 | 24.17 | 21.15 | 18.55 | 24.99 | 31.61 | 23.96 | 12.00 | 18.86 | 18.94 | 35.32 | 20.11 |
| Other | 4.91 | 3.69 | 5.80 | 4.47 | 6.90 | 3.63 | 6.59 | 1.03 | 3.31 | 2.67 | 2.48 | 2.39 |
| Mental illness characteristics |  |  |  |  |  |  |  |  |  |  |  |  |
| Mental disorder, (%) |  |  |  |  |  |  |  |  |  |  |  |  |
| Neuroses | 20.05 | 19.02 | 22.38 | 14.22 | 21.77 | 8.12 | 21.37 | 15.95 | 18.08 | 18.02 | 12.24 | 12.46 |
| Depression | 0.87 | 1.29 | 0.99 | 1.09 | 0.94 | 1.15 | 0.92 | 1.36 | 1.38 | 1.51 | 0.44 | 1.24 |
| Schizophrenia | 1.57 | 0.93 | 1.33 | 0.52 | 2.46 | 0.91 | 1.91 | 9.81 | 4.04 | 3.13 | 1.76 | 1.27 |
| Organic psychoses | 2.72 | 3.05 | 2.59 | 0.51 | 2.91 | 11.50 | 3.53 | 5.23 | 5.81 | 5.53 | 5.86 | 5.18 |
| Other psychoses | 4.07 | 3.69 | 4.02 | 2.39 | 5.76 | 4.23 | 5.35 | 10.88 | 8.63 | 7.24 | 4.89 | 3.78 |
| Alcoholism | 0.44 | 0.83 | 0.50 | 0.77 | 0.52 | 0.00 | 0.52 | 1.11 | 0.40 | 0.86 | 0.81 | 1.68 |
| Dementia | 3.92 | 4.52 | 4.14 | 1.14 | 4.21 | 4.87 | 4.16 | 3.90 | 7.33 | 7.62 | 8.26 | 8.30 |
| Mental related medications, (%) |  |  |  |  |  |  |  |  |  |  |  |  |
| Antipsychotics | 15.37 | 17.09 | 16.20 | 17.98 | 17.40 | 24.00 | 16.47 | 17.17 | 22.34 | 22.12 | 23.95 | 20.05 |
| Antidepressant | 16.33 | 14.92 | 20.83 | 19.51 | 17.62 | 9.53 | 17.25 | 12.54 | 19.14 | 16.95 | 15.05 | 13.57 |
| Benzodiazepines | 53.83 | 51.29 | 60.03 | 66.14 | 57.30 | 42.44 | 57.32 | 48.26 | 55.96 | 53.23 | 55.54 | 50.61 |
| Dementia medication | 3.81 | 3.47 | 3.90 | 4.14 | 3.58 | 0.29 | 3.95 | 4.50 | 6.03 | 5.24 | 4.11 | 4.56 |
| Comorbidity related to epilepsy |  |  |  |  |  |  |  |  |  |  |  |  |
| Ischemic stroke | 8.91 | 11.80 | 9.77 | 12.81 | 9.63 | 11.90 | 9.11 | 10.12 | 14.27 | 14.55 | 18.74 | 19.24 |
| Hemorrhagic stroke | 2.40 | 3.66 | 2.32 | 6.29 | 2.40 | 0.00 | 2.37 | 0.94 | 4.75 | 5.62 | 8.08 | 11.67 |
| Brain neoplasm | 1.58 | 2.09 | 1.44 | 3.84 | 1.50 | 0.41 | 1.52 | 1.05 | 2.27 | 2.69 | 0.90 | 2.23 |
| Other comorbid conditions, (%) |  |  |  |  |  |  |  |  |  |  |  |  |
| Hypertension | 34.74 | 36.30 | 41.27 | 40.38 | 36.83 | 39.55 | 36.45 | 32.08 | 36.92 | 37.54 | 43.99 | 43.81 |
| Osteoarthritis | 20.49 | 20.16 | 26.14 | 25.38 | 25.34 | 7.92 | 23.85 | 14.96 | 18.51 | 17.64 | 17.88 | 15.63 |
| Diabetes mellitus | 17.71 | 13.85 | 20.42 | 19.57 | 18.66 | 17.36 | 18.94 | 22.47 | 18.19 | 17.82 | 21.66 | 20.70 |
| Peptic ulcer | 15.09 | 13.58 | 18.97 | 18.76 | 17.54 | 4.77 | 17.57 | 15.39 | 15.38 | 15.18 | 16.91 | 15.09 |
| Dyslipidemia | 14.83 | 15.52 | 18.02 | 19.62 | 15.45 | 3.75 | 15.67 | 12.80 | 14.40 | 14.10 | 13.38 | 13.40 |
| Other cardiovascular diseases | 15.05 | 14.95 | 16.58 | 14.51 | 15.72 | 12.71 | 15.49 | 14.49 | 21.16 | 21.39 | 27.42 | 27.37 |
| Liver disorders | 10.64 | 11.09 | 11.75 | 17.04 | 11.27 | 3.29 | 11.16 | 17.03 | 11.42 | 8.74 | 13.94 | 9.86 |
| Fractures | 7.53 | 7.53 | 8.33 | 11.26 | 7.97 | 4.39 | 7.86 | 8.04 | 7.85 | 9.21 | 11.04 | 9.70 |
| Migraine | 2.45 | 1.85 | 2.33 | 4.00 | 2.55 | 4.21 | 3.70 | 2.81 | 2.30 | 3.08 | 1.79 | 1.31 |
| Parkinsonism | 1.38 | 2.02 | 1.54 | 1.49 | 1.68 | 6.55 | 1.42 | 0.53 | 3.69 | 3.27 | 3.30 | 3.15 |
| Pneumonia | 5.64 | 5.20 | 6.09 | 7.14 | 6.26 | 3.46 | 6.30 | 7.28 | 6.17 | 6.22 | 12.29 | 10.67 |
| COPD | 10.33 | 6.81 | 11.35 | 7.35 | 11.54 | 3.63 | 10.97 | 5.20 | 11.24 | 9.43 | 18.76 | 11.29 |
| Gastrointestinal bleeding | 1.97 | 1.46 | 2.43 | 2.93 | 2.21 | 3.89 | 2.13 | 1.25 | 1.98 | 2.71 | 2.91 | 4.52 |
| Neoplasm at site outside brain | 2.70 | 1.70 | 4.60 | 5.40 | 2.82 | 1.32 | 2.73 | 0.80 | 2.81 | 3.75 | 3.95 | 4.50 |
| Ischemic heart disease | 11.54 | 9.46 | 14.61 | 14.92 | 12.18 | 10.81 | 11.96 | 12.54 | 11.12 | 12.93 | 10.60 | 13.60 |
| Congestive heart failure | 3.31 | 4.66 | 3.86 | 7.50 | 3.47 | 3.23 | 3.36 | 0.40 | 3.56 | 4.66 | 5.40 | 5.86 |
| Eczema | 2.55 | 5.53 | 2.77 | 1.11 | 2.90 | 6.79 | 2.74 | 3.60 | 2.57 | 2.11 | 4.02 | 1.80 |
| Rheumatoid arthritis | 1.66 | 2.63 | 2.73 | 3.89 | 1.92 | 0.00 | 2.12 | 0.09 | 1.35 | 1.13 | 1.46 | 0.73 |
| Cerebral degeneration | 1.02 | 1.62 | 1.12 | 0.67 | 1.08 | 0.73 | 1.13 | 0.56 | 2.30 | 2.58 | 2.29 | 3.59 |
| Renal failure | 2.94 | 1.64 | 3.59 | 3.52 | 3.38 | 10.35 | 3.31 | 1.24 | 2.45 | 3.63 | 3.36 | 4.11 |
| Other renal disorders | 3.15 | 1.23 | 3.56 | 3.67 | 3.32 | 3.44 | 3.39 | 4.17 | 3.46 | 4.09 | 4.76 | 2.78 |
| Concomitant medications, (%) |  |  |  |  |  |  |  |  |  |  |  |  |
| NSAIDs | 71.72 | 75.54 | 75.15 | 80.99 | 74.27 | 71.30 | 74.92 | 77.26 | 70.38 | 68.26 | 66.49 | 65.22 |
| Antihistamines | 61.30 | 64.26 | 65.40 | 64.05 | 63.11 | 51.11 | 63.30 | 71.61 | 61.89 | 57.57 | 54.81 | 53.59 |
| Antibacterial agents | 60.43 | 57.19 | 62.32 | 63.77 | 62.04 | 56.01 | 62.65 | 56.26 | 59.87 | 61.54 | 63.18 | 62.33 |
| Bowel medications | 40.35 | 43.28 | 45.63 | 52.38 | 43.45 | 29.52 | 43.78 | 40.59 | 39.93 | 38.02 | 38.42 | 36.56 |
| COPD medications | 39.83 | 43.73 | 42.72 | 42.18 | 41.46 | 33.04 | 40.82 | 54.24 | 39.90 | 36.55 | 44.82 | 40.94 |
| Propulsive | 35.48 | 35.60 | 40.99 | 43.75 | 37.59 | 30.99 | 38.45 | 40.40 | 37.37 | 35.44 | 36.89 | 37.48 |
| Ophthalmics | 32.66 | 36.80 | 37.12 | 47.61 | 34.10 | 42.51 | 34.40 | 46.25 | 34.03 | 36.19 | 32.46 | 33.52 |
| GERD medications | 31.61 | 29.38 | 35.54 | 44.51 | 34.43 | 18.77 | 35.14 | 28.38 | 28.22 | 29.11 | 32.97 | 31.86 |
| Systemic steroid | 29.99 | 30.27 | 33.52 | 37.92 | 31.42 | 19.97 | 31.84 | 31.89 | 29.61 | 27.62 | 33.92 | 27.85 |
| Calcium channel blockers | 29.25 | 29.07 | 34.35 | 34.85 | 31.32 | 27.49 | 31.30 | 23.06 | 31.68 | 29.75 | 38.54 | 35.40 |
| Antiplatelets | 25.58 | 25.30 | 30.38 | 27.37 | 26.98 | 25.70 | 26.63 | 19.07 | 29.14 | 29.02 | 33.10 | 31.95 |
| Beta-blockers | 23.25 | 22.51 | 27.20 | 31.16 | 24.83 | 17.11 | 25.28 | 28.73 | 24.33 | 25.24 | 22.98 | 26.65 |
| RAS inhibitors | 22.84 | 21.72 | 26.37 | 26.18 | 24.32 | 18.41 | 23.65 | 19.65 | 24.30 | 24.38 | 29.05 | 26.72 |
| Diuretics | 18.82 | 20.06 | 23.85 | 28.83 | 20.56 | 12.39 | 20.47 | 14.63 | 19.30 | 20.27 | 25.19 | 25.30 |
| Antidiabetes | 15.51 | 13.86 | 17.81 | 18.23 | 16.35 | 13.46 | 16.64 | 16.09 | 16.18 | 15.61 | 18.89 | 18.36 |
| Anti-hemorrhagic agents | 13.01 | 13.03 | 13.29 | 13.55 | 13.51 | 8.28 | 12.90 | 10.43 | 12.94 | 11.95 | 14.81 | 15.39 |
| Lipid-lowering agents | 12.19 | 13.00 | 14.47 | 17.26 | 12.22 | 13.14 | 12.54 | 7.05 | 12.31 | 11.48 | 11.14 | 12.09 |
| Gout medications | 9.36 | 6.22 | 12.27 | 11.73 | 11.99 | 1.14 | 11.87 | 5.80 | 7.82 | 8.42 | 10.53 | 8.32 |
| Antihypertensive agents | 7.31 | 4.08 | 8.31 | 9.26 | 8.02 | 7.44 | 7.78 | 5.21 | 8.40 | 8.65 | 10.49 | 9.16 |
| Antiparkinson agents | 6.02 | 5.73 | 6.29 | 4.06 | 7.35 | 4.99 | 6.43 | 4.91 | 11.04 | 8.51 | 8.36 | 5.86 |
| Hormone agents | 5.83 | 6.12 | 5.60 | 5.77 | 5.86 | 5.71 | 6.07 | 2.03 | 5.19 | 4.58 | 3.49 | 2.58 |
| Glycosides | 3.30 | 2.65 | 3.87 | 3.94 | 3.68 | 3.23 | 3.59 | 3.38 | 3.44 | 3.76 | 4.86 | 5.12 |
| Antiarrhythmic drugs | 3.08 | 2.50 | 4.09 | 4.22 | 3.48 | 4.22 | 3.33 | 1.03 | 3.22 | 4.37 | 5.01 | 5.08 |
| Antimycotics | 2.86 | 1.48 | 2.89 | 3.69 | 2.84 | 0.26 | 2.90 | 0.89 | 1.82 | 1.88 | 3.25 | 2.12 |
| Antithrombotic agents | 1.15 | 1.41 | 0.76 | 3.53 | 0.80 | 3.89 | 0.78 | 0.22 | 0.82 | 2.45 | 1.18 | 2.79 |
| Thyroid medications | 0.82 | 1.91 | 0.92 | 1.71 | 1.03 | 0.26 | 0.95 | 0.41 | 0.87 | 1.78 | 0.62 | 1.31 |
| Abbreviation: CBZ, carbamazepine; OXC, oxcarbazepine; PHT, phenytoin; GBP gabapentin; TPM topiramate; LTG lamotrigine; VPA, valproic acid; GERD, Gastroesophageal reflux disease; COPD, chronic obstructive pulmonary disease; NSAIDs, non-steroidal anti-inflammatory drugs; RAS, renin-angiotensin system. | | | | | | | | | | | | |

| **Appendix Table 2.** Patient characteristics after matching by high-dimensional propensity score | | | | | | | | | | | | |
| --- | --- | --- | --- | --- | --- | --- | --- | --- | --- | --- | --- | --- |
|  | **Matched Cohort 1** | | **Matched Cohort 2** | | **Matched Cohort 3** | | **Matched Cohort 4** | | **Matched Cohort 5** | | **Matched Cohort 6** | |
|  | **CBZ** | **OXC** | **CBZ** | **GBP** | **CBZ** | **LTG** | **CBZ** | **TPM** | **CBZ** | **VPA** | **CBZ** | **PHT** |
| Number of patients | 611 | 611 | 320 | 320 | 106 | 106 | 131 | 131 | 1072 | 1072 | 1077 | 1077 |
| Age, mean , y | 46.47 | 46.58 | 61.48 | 60.37 | 37.98 | 39.92 | 45.89 | 46.34 | 51.02 | 53.15 | 53.54 | 55.00 |
| Standard deviation, y | 19.71 | 19.88 | 15.65 | 16.54 | 17.80 | 18.28 | 18.96 | 19.78 | 20.43 | 20.98 | 20.44 | 20.43 |
| Patient distribution by age, y (%) |  |  |  |  |  |  |  |  |  |  |  |  |
| 18-34 | 34.04 | 34.70 | 4.38 | 7.50 | 50.94 | 45.28 | 34.35 | 33.59 | 26.77 | 24.72 | 22.10 | 22.19 |
| 35-50 | 22.91 | 21.77 | 17.81 | 18.75 | 29.25 | 28.30 | 24.43 | 25.95 | 20.34 | 19.31 | 19.96 | 18.38 |
| 50-65 | 19.15 | 20.79 | 30.94 | 31.56 | 6.60 | 12.26 | 23.66 | 20.61 | 21.74 | 21.46 | 22.01 | 22.75 |
| >65 | 23.90 | 22.75 | 46.88 | 42.19 | 13.21 | 14.15 | 17.56 | 19.85 | 31.16 | 34.51 | 35.93 | 36.68 |
| Gender |  |  |  |  |  |  |  |  |  |  |  |  |
| Male (%) | 54.66 | 52.05 | 57.81 | 55.31 | 51.89 | 33.02 | 42.75 | 44.27 | 53.26 | 55.50 | 56.08 | 53.48 |
| Index year, (%), y |  |  |  |  |  |  |  |  |  |  |  |  |
| 2005 | 13.91 | 14.08 | 12.81 | 14.06 | 15.09 | 16.04 | 24.43 | 19.85 | 23.41 | 22.76 | 26.37 | 26.65 |
| 2006 | 18.49 | 18.66 | 21.25 | 20.94 | 18.87 | 19.81 | 27.48 | 16.79 | 25.65 | 24.81 | 27.30 | 24.05 |
| 2007 | 21.28 | 20.62 | 22.81 | 22.19 | 20.75 | 18.87 | 16.79 | 25.95 | 18.38 | 19.87 | 17.73 | 18.29 |
| 2008 | 24.22 | 24.39 | 22.50 | 22.19 | 19.81 | 17.92 | 15.27 | 18.32 | 17.63 | 17.82 | 16.62 | 15.51 |
| 2009 | 22.09 | 22.26 | 20.63 | 20.63 | 25.47 | 27.36 | 16.03 | 19.08 | 14.93 | 14.74 | 11.98 | 15.51 |
| NHI premium levels, NT$ (%) |  |  |  |  |  |  |  |  |  |  |  |  |
| >25,000 | 33.55 | 41.41 | 30.63 | 34.06 | 20.75 | 39.62 | 38.93 | 32.82 | 30.69 | 27.61 | 29.90 | 24.70 |
| 15,000-25,000 | 51.88 | 48.12 | 56.25 | 55.94 | 69.81 | 50.00 | 51.15 | 56.49 | 56.62 | 56.72 | 56.45 | 59.42 |
| <15,000 | 14.57 | 10.47 | 13.13 | 10.00 | 9.43 | 10.38 | 9.92 | 10.69 | 12.69 | 15.67 | 13.65 | 15.88 |
| Dosages of AED, |  |  |  |  |  |  |  |  |  |  |  |  |
| Mean, PDD/DDD ratio | 0.42 | 0.46 | 0.28 | 0.27 | 0.28 | 0.25 | 0.24 | 0.23 | 0.42 | 0.40 | 0.48 | 0.49 |
| Standard deviation, PDD/DDD ratio | 0.24 | 0.23 | 0.14 | 0.16 | 0.16 | 0.18 | 0.12 | 0.15 | 0.24 | 0.33 | 0.26 | 0.21 |
| Level of hospital, (%) |  |  |  |  |  |  |  |  |  |  |  |  |
| Medical center | 46.81 | 49.75 | 39.69 | 40.31 | 58.49 | 51.89 | 49.62 | 51.15 | 34.05 | 29.94 | 30.73 | 33.80 |
| Regional | 38.95 | 36.01 | 45.00 | 47.50 | 27.36 | 33.02 | 29.01 | 32.06 | 39.93 | 42.54 | 38.63 | 32.96 |
| District | 13.91 | 13.91 | 12.19 | 10.31 | 13.21 | 13.21 | 17.56 | 14.50 | 22.76 | 24.91 | 25.91 | 28.32 |
| Other | 0.33 | 0.33 | 3.13 | 1.88 | 0.94 | 1.89 | 3.82 | 2.29 | 3.26 | 2.61 | 4.74 | 4.92 |
| Mental illness characteristics |  |  |  |  |  |  |  |  |  |  |  |  |
| Mental disorder, (%) |  |  |  |  |  |  |  |  |  |  |  |  |
| Neuroses | 17.18 | 17.68 | 28.75 | 18.44 | 21.70 | 15.09 | 19.08 | 16.03 | 19.22 | 20.43 | 17.46 | 21.26 |
| Depression | 0.82 | 1.47 | 1.88 | 2.50 | 0.94 | 2.83 | 0.76 | 3.05 | 1.03 | 1.49 | 0.56 | 1.76 |
| Schizophrenia | 1.31 | 1.31 | 1.56 | 0.94 | 7.55 | 1.89 | 3.05 | 3.82 | 1.87 | 2.33 | 1.39 | 1.49 |
| Organic psychoses | 2.13 | 3.44 | 2.81 | 0.63 | 0.94 | 7.55 | 6.87 | 5.34 | 3.54 | 3.45 | 3.16 | 5.48 |
| Other psychoses | 3.11 | 4.58 | 4.69 | 4.06 | 15.09 | 13.21 | 7.63 | 6.11 | 5.04 | 5.22 | 4.55 | 4.64 |
| Alcoholism | 0.16 | 0.98 | 0.63 | 0.94 | 0.00 | 0.00 | 0.76 | 2.29 | 0.47 | 0.84 | 0.65 | 0.84 |
| Dementia | 2.62 | 3.60 | 5.31 | 1.88 | 0.00 | 4.72 | 2.29 | 2.29 | 5.04 | 5.13 | 5.01 | 7.61 |
| Mental related medications, (%) |  |  |  |  |  |  |  |  |  |  |  |  |
| Antipsychotics | 14.08 | 17.68 | 18.75 | 18.44 | 19.81 | 27.36 | 11.45 | 19.85 | 18.10 | 19.59 | 16.99 | 21.54 |
| Antidepressant | 14.24 | 13.75 | 27.50 | 28.75 | 11.32 | 16.98 | 16.03 | 12.98 | 17.63 | 16.60 | 15.97 | 18.48 |
| Benzodiazepines | 46.97 | 45.99 | 65.94 | 68.13 | 51.89 | 41.51 | 52.67 | 43.51 | 54.38 | 54.29 | 55.71 | 56.64 |
| Dementia medication | 2.95 | 3.27 | 3.44 | 5.94 | 0.94 | 1.89 | 3.05 | 3.05 | 3.64 | 4.20 | 3.62 | 4.55 |
| Comorbidity related to epilepsy |  |  |  |  |  |  |  |  |  |  |  |  |
| Ischemic stroke | 6.55 | 10.64 | 11.25 | 13.75 | 4.72 | 7.55 | 6.11 | 10.69 | 10.63 | 11.94 | 11.42 | 12.72 |
| Hemorrhagic stroke | 2.62 | 3.93 | 2.50 | 5.94 | 0.94 | 0.00 | 2.29 | 3.05 | 3.08 | 3.36 | 3.06 | 3.71 |
| Brain neoplasm | 1.64 | 2.62 | 1.25 | 2.50 | 1.89 | 1.89 | 1.53 | 3.05 | 1.77 | 1.68 | 1.58 | 2.88 |
| Other comorbid conditions, (%) |  |  |  |  |  |  |  |  |  |  |  |  |
| Hypertension | 27.99 | 29.95 | 50.94 | 47.81 | 20.75 | 23.58 | 23.66 | 29.01 | 35.54 | 37.31 | 39.37 | 40.39 |
| Osteoarthritis | 10.47 | 11.62 | 27.81 | 33.44 | 11.32 | 6.60 | 8.40 | 8.40 | 19.78 | 21.27 | 23.03 | 26.93 |
| Diabetes mellitus | 13.75 | 12.11 | 25.94 | 24.69 | 7.55 | 12.26 | 16.03 | 14.50 | 18.94 | 18.38 | 19.87 | 19.96 |
| Peptic ulcer | 9.66 | 10.64 | 24.69 | 20.31 | 11.32 | 6.60 | 16.79 | 16.79 | 16.60 | 15.21 | 17.64 | 17.27 |
| Dyslipidemia | 11.95 | 12.93 | 25.31 | 21.56 | 6.60 | 8.49 | 19.85 | 12.21 | 15.11 | 14.74 | 14.76 | 16.25 |
| Other cardiovascular diseases | 11.78 | 13.91 | 17.19 | 17.50 | 4.72 | 10.38 | 9.16 | 13.74 | 16.60 | 19.31 | 18.38 | 21.54 |
| Liver disorders | 9.33 | 7.20 | 14.69 | 16.25 | 8.49 | 7.55 | 12.21 | 16.79 | 11.38 | 9.89 | 11.33 | 11.05 |
| Fractures | 6.06 | 6.22 | 10.00 | 8.44 | 2.83 | 2.83 | 8.40 | 5.34 | 7.56 | 10.07 | 8.17 | 8.91 |
| Migraine | 2.29 | 2.13 | 2.19 | 3.13 | 3.77 | 3.77 | 7.63 | 6.11 | 2.71 | 3.26 | 2.41 | 1.86 |
| Parkinsonism | 1.15 | 1.96 | 0.94 | 2.50 | 0.94 | 1.89 | 0.00 | 1.53 | 1.68 | 1.68 | 1.76 | 1.58 |
| Pneumonia | 4.26 | 4.09 | 5.94 | 6.25 | 3.77 | 2.83 | 3.05 | 4.58 | 5.78 | 5.41 | 6.96 | 7.99 |
| COPD | 6.22 | 5.40 | 10.00 | 8.13 | 7.55 | 1.89 | 1.53 | 5.34 | 10.82 | 10.45 | 12.72 | 14.86 |
| Gastrointestinal bleeding | 1.80 | 1.64 | 3.75 | 2.50 | 0.00 | 1.89 | 1.53 | 3.82 | 2.24 | 2.43 | 2.41 | 3.90 |
| Neoplasm at site outside brain | 2.29 | 1.96 | 5.00 | 7.50 | 2.83 | 1.89 | 2.29 | 1.53 | 2.71 | 3.45 | 3.34 | 4.92 |
| Ischemic heart disease | 9.17 | 9.00 | 19.69 | 22.19 | 8.49 | 5.66 | 9.92 | 8.40 | 11.57 | 14.18 | 11.70 | 14.67 |
| Congestive heart failure | 2.13 | 2.45 | 5.00 | 7.50 | 0.94 | 0.94 | 0.76 | 0.76 | 3.45 | 4.38 | 3.99 | 5.94 |
| Eczema | 1.96 | 1.96 | 3.44 | 2.19 | 0.00 | 3.77 | 1.53 | 1.53 | 2.61 | 2.43 | 2.97 | 2.32 |
| Rheumatoid arthritis | 0.82 | 0.82 | 2.81 | 3.75 | 0.94 | 0.00 | 3.05 | 0.00 | 1.40 | 1.21 | 1.21 | 0.74 |
| Cerebral degeneration | 0.82 | 1.80 | 0.94 | 1.25 | 0.00 | 0.94 | 1.53 | 0.76 | 1.40 | 1.77 | 1.30 | 2.23 |
| Renal failure | 1.80 | 1.64 | 4.69 | 5.00 | 4.72 | 3.77 | 2.29 | 3.05 | 2.89 | 3.92 | 3.62 | 5.01 |
| Other renal disorders | 2.29 | 1.31 | 5.00 | 5.31 | 0.94 | 0.94 | 3.05 | 3.05 | 3.17 | 5.04 | 3.62 | 2.69 |
| Concomitant medications, (%) |  |  |  |  |  |  |  |  |  |  |  |  |
| NSAIDs | 69.39 | 70.38 | 79.06 | 83.75 | 68.87 | 67.92 | 76.34 | 69.47 | 72.11 | 71.08 | 72.14 | 72.89 |
| Antihistamines | 58.10 | 64.48 | 66.25 | 65.00 | 58.49 | 53.77 | 69.47 | 65.65 | 62.22 | 59.61 | 60.72 | 62.40 |
| Antibacterial agents | 57.45 | 60.23 | 62.81 | 68.13 | 53.77 | 51.89 | 58.78 | 53.44 | 60.91 | 65.11 | 61.37 | 65.83 |
| Bowel medications | 36.17 | 37.15 | 49.38 | 50.63 | 42.45 | 34.91 | 44.27 | 39.69 | 41.79 | 40.67 | 42.15 | 40.11 |
| COPD medications | 35.35 | 37.81 | 46.88 | 42.19 | 32.08 | 25.47 | 41.22 | 39.69 | 40.76 | 36.19 | 41.32 | 46.24 |
| Propulsive | 33.06 | 30.11 | 43.44 | 45.63 | 31.13 | 32.08 | 40.46 | 31.30 | 36.85 | 35.54 | 36.95 | 39.37 |
| Ophthalmics | 29.46 | 34.53 | 42.50 | 51.88 | 26.42 | 35.85 | 43.51 | 36.64 | 33.96 | 37.59 | 32.68 | 37.23 |
| GERD medications | 25.37 | 24.55 | 40.31 | 41.25 | 25.47 | 19.81 | 34.35 | 27.48 | 31.34 | 34.24 | 33.61 | 32.96 |
| Systemic steroid | 24.88 | 25.86 | 35.63 | 40.63 | 21.70 | 12.26 | 31.30 | 27.48 | 30.32 | 28.64 | 32.31 | 30.45 |
| Calcium channel blockers | 23.57 | 23.08 | 39.38 | 42.19 | 16.98 | 16.98 | 20.61 | 22.90 | 30.04 | 29.01 | 33.05 | 34.26 |
| Antiplatelets | 18.82 | 22.59 | 39.06 | 31.88 | 11.32 | 15.09 | 20.61 | 17.56 | 26.68 | 28.73 | 29.43 | 29.81 |
| Beta-blockers | 20.62 | 22.09 | 33.13 | 35.31 | 22.64 | 18.87 | 32.06 | 28.24 | 24.53 | 25.75 | 24.23 | 27.86 |
| RAS inhibitors | 17.35 | 18.82 | 35.63 | 30.00 | 8.49 | 13.21 | 12.98 | 12.98 | 23.79 | 23.60 | 26.18 | 26.00 |
| Diuretics | 13.58 | 14.40 | 27.81 | 29.38 | 12.26 | 8.49 | 14.50 | 14.50 | 19.12 | 20.06 | 22.28 | 24.98 |
| Antidiabetes | 12.27 | 11.29 | 22.19 | 22.50 | 7.55 | 9.43 | 12.98 | 12.21 | 16.51 | 16.51 | 17.55 | 17.55 |
| Anti-hemorrhagic agents | 11.95 | 12.27 | 15.63 | 16.88 | 10.38 | 5.66 | 10.69 | 11.45 | 13.71 | 11.66 | 13.56 | 14.30 |
| Lipid-lowering agents | 9.98 | 11.62 | 20.00 | 19.69 | 6.60 | 10.38 | 10.69 | 10.69 | 12.69 | 11.29 | 12.44 | 14.21 |
| Gout medications | 3.76 | 5.24 | 15.63 | 14.38 | 8.49 | 3.77 | 9.92 | 6.87 | 9.89 | 11.10 | 11.42 | 9.66 |
| Antihypertensive agents | 6.06 | 4.26 | 9.38 | 13.44 | 3.77 | 3.77 | 6.87 | 4.58 | 7.28 | 8.68 | 8.64 | 8.26 |
| Antiparkinson agents | 5.56 | 5.07 | 5.63 | 7.19 | 10.38 | 4.72 | 6.11 | 6.11 | 6.81 | 6.72 | 6.50 | 5.48 |
| Hormone agents | 6.71 | 6.55 | 4.38 | 5.00 | 6.60 | 9.43 | 8.40 | 3.82 | 5.41 | 5.69 | 4.74 | 4.27 |
| Glycosides | 1.47 | 1.80 | 4.38 | 4.06 | 1.89 | 0.94 | 0.76 | 1.53 | 3.36 | 4.29 | 3.71 | 4.55 |
| Antiarrhythmic drugs | 2.45 | 2.29 | 4.69 | 4.38 | 0.00 | 2.83 | 2.29 | 1.53 | 3.36 | 4.76 | 3.71 | 4.36 |
| Antimycotics | 2.95 | 1.64 | 1.88 | 3.44 | 2.83 | 0.94 | 3.05 | 2.29 | 2.15 | 2.24 | 2.51 | 1.76 |
| Antithrombotic agents | 1.15 | 1.15 | 1.25 | 3.13 | 0.00 | 1.89 | 0.00 | 0.76 | 0.84 | 1.87 | 1.02 | 2.23 |
| Thyroid medications | 0.49 | 1.96 | 0.94 | 2.19 | 1.89 | 0.00 | 0.76 | 2.29 | 0.93 | 2.15 | 0.74 | 1.95 |
| Abbreviation: CBZ, carbamazepine; OXC, oxcarbazepine; PHT, phenytoin; GBP gabapentin; TPM topiramate; LTG lamotrigine; VPA, valproic acid; GERD, Gastroesophageal reflux disease; COPD, chronic obstructive pulmonary disease; NSAIDs, non-steroidal anti-inflammatory drugs; RAS, renin-angiotensin system. | | | | | | | | | | | | |

**Appendix Figure 1.** Secondary analyses of persistence comparisons among antiepileptic drugs by treatment changes using inverse probability weighting and matching with high-dimensional propensity score.


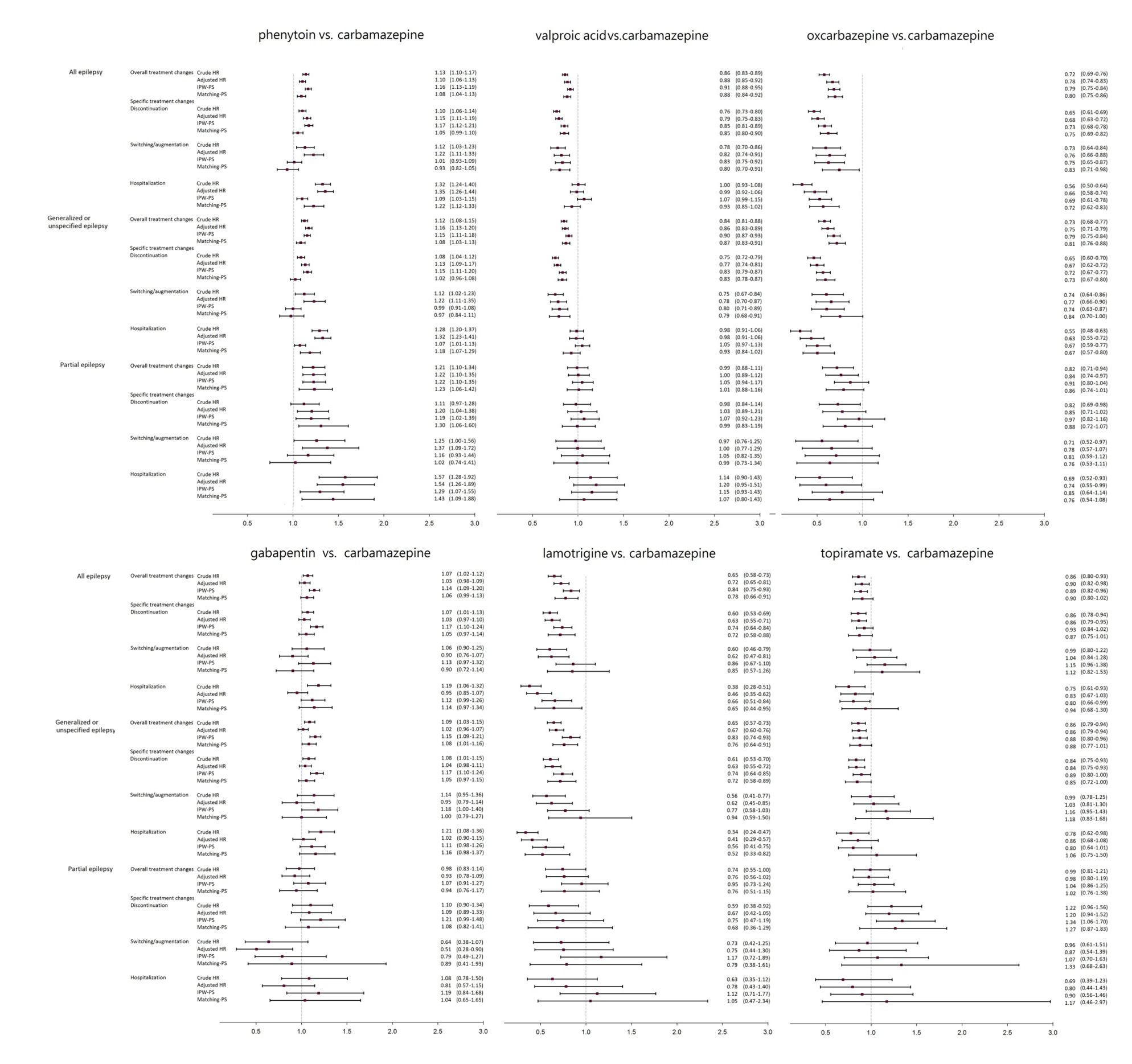

Supplement: Supplemental Digital Content [file medi-95-e4481-s001.doc]
